# Astrocytic gap junction inhibition by carbenoxolone enhances the protective effects of ischemic preconditioning following cerebral ischemia

**DOI:** 10.1186/s12974-018-1230-5

**Published:** 2018-07-05

**Authors:** Di Ma, Liangshu Feng, Yingying Cheng, Meiying Xin, Jiulin You, Xiang Yin, Yulei Hao, Li Cui, Jiachun Feng

**Affiliations:** 1 Department of Neurology and Neuroscience center, The First Hospital of Jilin University, Changchun 130021, Jilin Province, People’s Republic of China; 2 http://www.jdyy.cn/

**Keywords:** Cerebral ischemic preconditioning, Ischemia reperfusion, Reactive oxygen species, Oxygen-glucose deprivation, Gap junction, Connexin 43, Carbenoxolone, Glutamate, Astrocyte, Inflammation

## Abstract

**Background:**

Stroke is the second leading cause of death worldwide and the most common cause of adult-acquired disability in many nations. Thus, attenuating the damage after ischemic injury and improving patient prognosis are of great importance. We have indicated that ischemic preconditioning (IP) can effectively reduce the damage of ischemia reperfusion and that inhibition of gap junctions may further reduce this damage. Although we confirmed that the function of gap junctions is closely associated with glutamate, we did not investigate the mechanism. In the present study, we aimed to clarify whether the blockade of cellular communication at gap junctions leads to significant reductions in the levels of glutamate released by astrocytes following cerebral ischemia.

**Methods:**

To explore this hypothesis, we utilized the specific blocking agent carbenoxolone (CBX) to inhibit the opening and internalization of connexin 43 channels in an in vitro model of oxygen-glucose deprivation/re-oxygenation (OGD/R), following IP.

**Results:**

OGD/R resulted in extensive astrocytic glutamate release following upregulation of hemichannel activity, thus increasing reactive oxygen species (ROS) generation and subsequent cell death. However, we observed significant increases in neuronal survival in neuron-astrocyte co-cultures that were subjected to IP prior to OGD/R. Moreover, the addition of CBX enhanced the protective effects of IP during the re-oxygenation period following OGD, by means of blocking the release of glutamate, increasing the level of the excitatory amino acid transporter 1, and downregulating glutamine expression.

**Conclusions:**

Our results suggest that combined use of IP and CBX represents a novel therapeutic strategy to attenuate damage from cerebral ischemia with minimal adverse side effects.

## Background

Recent studies have increasingly focused on the therapeutic potential of ischemic preconditioning (IP) in attenuating structural and functional deficits following ischemia reperfusion [1]. IP has been widely adopted as a clinical strategy to protect the brain from subsequent, more serious ischemia-reperfusion insults [2]; this process involves the induction of brief periods of subthreshold ischemia to prevent or attenuate severe ischemic injury as a result of subsequent, prolonged periods of ischemia [3, 4]. Notably, several clinical trials have confirmed that such preconditioning strategies can attenuate the pathophysiological consequences of ischemia-reperfusion injury prior to cardiac bypass surgery [5, 6].

Previously, we reported that IP can effectively reduce the infarct area in the brain; in order to explore the mechanism associated with this neuroprotection, we investigated the function of gap junctions during the process of ischemia reperfusion [7]. Hemichannels, which can be found on the astrocyte membrane, are composed of connexin 43 (Cx43), which allows the outflow of small molecules from astrocytes under some pathological conditions. Gap junctions (GJs) are composed of hemichannels found at the corresponding position on the cell membrane where adjacent cells contact each other, thus forming channels which allow GJ-mediated intercellular communication (GJIC). This enables the synchronized information transfer between adjacent astrocytes, as well as metabolic substrate exchange and ion balance [8]. The regulation of the internal environment [9–11] is a function of astrocytes; thus, Cx43, found both on the mitochondria and the cell membrane of astrocytes, as well as the hemichannels and gap junctions it forms, might also play complex roles in ischemic injury [12, 13].

We previously confirmed the protective roles of mitochondrial Cx43 (mtCx43) during cerebral ischemia-reperfusion injury via protein kinase C activation [14]. In addition, salvianolic acids represent a potential treatment option for cerebral infarction by upregulating mtCx43 through the PI3K/AKT (phosphatidylinositol-4,5-bisphosphate 3-kinase/protein kinase B) pathway [14, 15]. Combined with the known mechanism for ischemia-reperfusion insults, we suspect that mitochondria may play a key role in ischemia-reperfusion insults and neuroprotection. Mitochondria directly use oxygen molecules to produce energy, while oxygen molecules undergoing the relevant reactions produce very potent intermediates (reactive oxygen species, ROS), which cause damage to organisms (known as oxygen toxicity) [16]. In this context, we believe that the neuroprotective effect of IP may be closely related to the role of ROS.

Recent studies have revealed that GJs may enable the spread of toxic or oxidative factors, such as excitatory amino acids, or induce calcium overload [17]. Excitatory amino acids, such as glutamate, promote inflammatory responses involving microglia, dendritic cells, and other antigen-presenting cells [18], while a number of pathological conditions lead to the release of glutamate, through GJs, by microglia and astrocytes [19]. Physiologically, glutamate is cleared predominantly by glial cells through the activity of highly efficient excitatory amino acid transporters (EAAT1 and EAAT2) and efficiently recycled in the glutamate/glutamine metabolic cycle [20]. Therefore, glial cells are not only responsible for protecting neurons from the deleterious effects of elevated glutamate levels but are also the principal source of synaptic glutamate release [21]. Under pathological conditions, the release of glutamate by astrocytes is likely to induce the expansion of the infarct and surrounding ischemic penumbra.

However, Cx43 might be harmful during ischemia-reperfusion according to most reports [22], and some studies are not supportive of our previous results, which may relate to the use of different in vivo and in vitro models, different mechanisms during different stages of cerebral ischemia and reperfusion, and the expression of other proteins associated with the formation of gap junctions, such as Cx30 [23–25]. We hypothesize that Cx43-containing GJs might be a “double-edged sword,” playing different (sometimes opposing) roles in a variety of injuries and diseases, or even at different times during the same injury. Healthy cells might rescue dying cells by transferring essential metabolites via these Cx43 GJs. Alternatively, dying astrocytes might compromise the survival of neighboring cells via these junctions, thereby promoting the propagation of cell death [26].

In the present study, we aimed to clarify whether the blockade of cellular communication via the GJs leads to significant reductions in the levels of glutamate released by astrocytes, following cerebral ischemia. We hypothesized that astrocytes are critically involved in promoting ischemic injury through GJs, which involves glutamate and ROS. To explore this hypothesis, we utilized the specific blocking agent carbenoxolone (CBX) [27] to inhibit Cx43 opening and detect the extent of cell damage and glutamate/ROS-related indicators. To explore the mechanism of IP, we further used an in vitro model of IP and combined it with oxygen-glucose deprivation/re-oxygenation (OGD/R).

## Methods

All animals were bred at Jilin University, and all animal studies were conducted according to the guidelines of the National Regulation of China for Care and Use of Laboratory Animals. Tissue harvesting was performed at Jilin University.

### Cell culture

#### Astrocyte culture

Astrocyte cultures were prepared according to a previous report [28]. In brief, primary astrocyte cultures were prepared from the cortex of 1-day-old Wistar rats which were bred at Jilin University; the cortices were dissected and grinded and then passed through a 40-μm cell strainer. Cells were collected after centrifugation at 250*g* for 10 min then resuspended and seeded in 75-cm^2^ culture flasks at a concentration of 1 × 10^5^ cells/cm^2^. Cells were passaged at a 1:2 ratio when they reached approximately 80% confluence; cells were passaged three times to purify astrocytes. The purity of astrocytes was verified by glial fibrillary acidic protein (GFAP) (astrocytes specific marker) and CD11b-FITC (microglia specific marker) staining. The final cultures contained > 95% astrocytes and < 5% microglia.

#### Primary neuron culture

Primary neuron cultures were prepared from cortices of embryonic day 17 (E17) Wistar rat embryos. Briefly, cortical fragments were dissociated into single cells in dissociation solution (Sumitomo Bakelite; Akita, Japan) and resuspended in nerve culture medium (Sumitomo Bakelite). Neurons were plated onto 12-mm polyethyleneimine (PEI)-coated glass coverslips (Asahi Techno Glass; Chiba, Japan) at a density of 5 × 10^4^ cells/well in 24-well plates and incubated at 37 °C in a humidified atmosphere containing 95% O2/5% CO2. The purity of the cultures was > 95%, as determined via neuronal nuclei (NeuN)-specific immunostaining.

#### Neuron-astrocyte co-culture

To produce neuron-astrocyte co-cultures, purified astrocytes that had been passaged twice were seeded and grown on 12-mm PEI-coated cover glasses in 24-well plates. After the astrocytes had reached approximately 80% confluence, neuronal cells (5 × 10^4^ cells/well) resuspended in 10 μL neuron medium were added to the astrocyte cultures in 24-well plates.

### IP and OGD/R model

OGD was performed for 2 h, followed by the reintroduction of oxygen and glucose. We measured changes in outcomes of interest at 1, 3, or 6 h after reintroduction. In the IP + OGD/R group, cell cultures were subjected to transient OGD for 30 min, 1 day prior to the 2 h of OGD, followed by the reintroduction of oxygen and glucose. The duration of IP was chosen based on Nikiforou et al. [28]. Depending on the purpose of the experiment, various drugs were added on the basis of OGD/R or IP, and the different experimental groups are shown in the result section.

### Drug administration

CBX and 6-diazo-5-oxo-l-norleucine (DON) were purchased from Sigma-Aldrich. CBX was used to block GJs and hemichannels, while DON was used to inhibit glutaminase (Gls) and reduce glutamate production [29]. CBX was used at a concentration of 20 μM, as in our previous study [27]. The final concentration of DON was 100 μM [19]. The final concentrations of anti-tumor necrosis factor-α (TNF-α) antibody (aTNF) and anti-interleukin (IL)-1 receptor antibody (IL-1ra) were 10 μg/mL. All drugs were added to the cell culture 30 min prior to the start of OGD.

### Cell counting

The numbers of astrocytes and neurons were determined via double staining with a neuronal (microtubule-associated protein II, MAP-II) and activated astrocyte marker (GFAP) using laser confocal microscopy (Olympus Fluoview FV1000). Cell counting was conducted using WCIF Image J.

### Quantitation for IL-1β, TNF-α, glutamate, and NO

After 6 h of re-oxygenation, the supernatants from astrocyte cultures were assessed using enzyme-linked immunosorbent assay kits for TNF-α and IL-1β (BD Pharmingen; Franklin Lakes, NJ). Nitric oxide (NO) and glutamate were measured using commercially available kits (Nanjing Jiancheng Bioengineering Institute, China), according to the manufacturer’s instructions.

### Measurement of ROS

Live cell staining for ROS was performed in astrocytes or astrocyte-neuron co-cultures using the ROS sensor reagent CellROX™ Deep Red (red) and an antibody for the astrocyte cell surface marker glutamate-aspartate transporter (GLAST, green). Confocal microscope (Carl Zeiss LSM Pascal 5) was used to capture the fluorescent signals generated intracellularly. The images were processed by WCIF Image J.

We also performed quantitative measurements of ROS synthesis using the iMark™ microplate reader (BIO-RAD). Astrocytes were prepared as a single cell suspension and incubated with fresh medium containing 10 μM DCFH-DA for 30 min at 37 °C in a 5% CO_2_ incubator. After incubation, cells were washed twice with 1× phosphate-buffered saline and then loaded onto dark-light 96-well plates at a concentration of 5 × 10^4^ cells/mL (0.1 mL/well). After 2 days of plating, OGD was performed for 2 h, followed by reoxygenation for 6 h; then, the absorbance at 485 nm was detected using a fluorescence microplate reader. Five replicates were performed for each group.

### Ethidium bromide uptake

For dye uptake experiments, astrocytes cultured on coverslips (14 mm in diameter; Gassalem; Limeil-Brévannes, France) were exposed to 0.5 μM ethidium bromide (Molecular Probes; Eugene, OR) for 10 min at 37 °C. The cells were then washed with Hank’s balanced salt solution (in mM: NaCl, 137; KCl, 5.4; Na_2_HPO_4_, 0.34; KH_2_PO_4_, 0.44; pH 7.4) supplemented with 1.2 mM CaCl_2_. To visualize ethidium (Etd) uptake, coverslips were mounted in Fluoromount (Southern Biotech; Birmingham, AL) and examined via epifluorescence (518 nm excitation and 605 nm emission), using an inverted microscope (Daiphot Nikon) equipped with a charge-coupled device camera (Nikon). Images were analyzed using software provided by the manufacturer (Lucia-Nikon). Images of Etd uptake were analyzed by counting the number of Etd-positive cells per field using the WCIF ImageJ software (v2.1.4.7 NIH software; Scion Corp.; Frederik, MA). For each experimental condition, ten microscopic fields per coverslip were arbitrarily selected and averaged. Data are expressed as the number of Etd-positive cells per field.

### Western blot analysis

Western blot analysis was conducted as previously described [14]. Briefly, cells were collected in PBS and centrifuged, and the supernatant was removed. The remaining pellet was dissolved in lysis buffer, and the cell lysates were quantified with Bio-Rad protein assay solution. The homogenates (20 μg protein) were separated by SDS-PAGE. After transfer, membranes were blocked in 5% skim milk (or BSA) and incubated overnight at 4 °C with the primary antibodies. The primary antibodies (diluted in Tris-buffered saline with Tween) included rabbit-derived anti-Cx43 (1:1000; Cell signaling technology), anti-p-Cx43 (Ser368; 1:1000; Abcam), mouse-derived anti-β-actin, and anti-GFAP (1:1000; Abcam). After incubation with primary antibodies, the membranes were washed and incubated with horseradish peroxidase-conjugated secondary antibodies for 1 h. Immunoreactive proteins were detected by ECL and analyzed by an Odyssey infrared imaging system (LiCor, Lincoln, NE, USA). Protein bands were quantified using Image J software (http://imagej.nih.gov/ij/), and intensity was expressed relative to the control value.

### Real-time PCR

Total RNA was extracted from the cells using the RNA extraction kit (Nachuankeji, China), according to the manufacturer’s instructions. A total of 1 μg of total RNA was transcribed with oligo(dT)_18_ primer and reverse transcriptase using the RevertAid First Strand cDNA Synthesis Kit (Thermo Scientific; Waltham, MA). Real-time PCR, targeting Gls, was performed using the powerUpTM SYBRTM Green Master Mix (Applied Biosystems; Foster City, CA) in a final volume of 10 μL, according to the manufacturer’s instructions. Sequences of the primers were as follows: 5′-GGAAAGCTGTGGCGTGAT-3′ [glyceraldehyde 3-phosphate dehydrogenase (GA DPH) forward] and 5′-AAGGTGGAAGAATGGGAGTT-3′ (GADPH reverse); 5′-CACC TCTGGGCATCCTCTTC-3′ (EAAT1 forward) and 5′-CCCCCAATCACACCCATATC-3′ (EAAT1 reverse); and 5′-CAGAACAGCCCTGCATGTTGCTG-3′ (Gls forward) and 5′-CCACCTG TCCTTGGGGAAGGGGT-3′ (Gls reverse). After 40 cycles of amplification (Uracil-DNA glycosylase activation at 50 °C for 2 min, Dual-Lock™ DNA polymerase release at 95 °C for 2 min, denaturation at 95 °C for 15 s, anneal/extend at 60 °C for 1 min), the CT values of each sample were read by the SteponePlus Real-Time PCR system (Applied Biosystems).

### Statistical analysis

The statistical significance of the results was assessed using one-way analysis of variance, followed by post hoc Tukey tests. All analyses were performed using GraphPad Prism, version 6.0 (GraphPad Software, La Jolla, CA).

## Results

### CBX enhances the protective effects of IP after 2 h of OGD or 6 h of re-oxygenation

We supposed that GJs may exert a unique pathophysiological role during cerebral ischemia. To explore this hypothesis, we utilized the specific blocking agent CBX to inhibit Cx43 opening and detect the extent of cell damage. In addition, to explore the mechanism of IP, we performed in vitro IP before OGD/R.

**Fig. 1:**
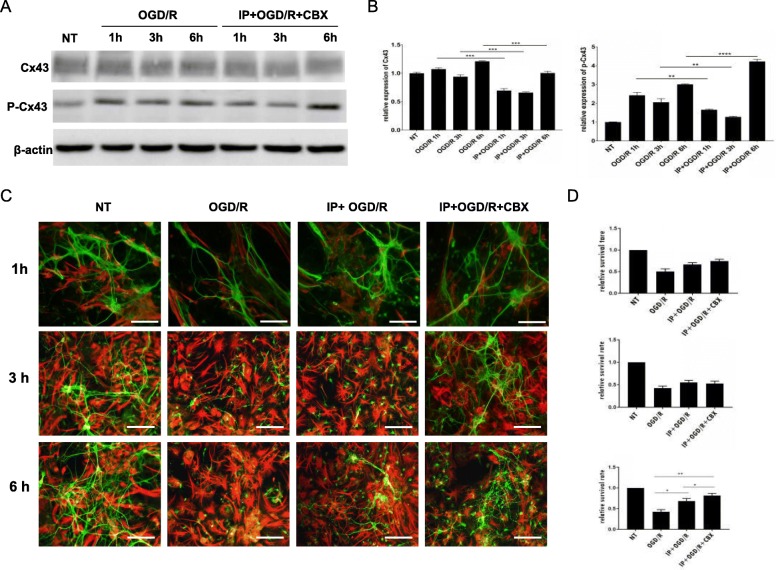
Changes in cell survival in the four groups 1, 3, and 6 h after re-oxygenation. **a** Western blot analysis of Cx43 and p-Cx43 in astrocytes 1, 3, and 6 h after re-oxygenation. **b** Relative expression of Cx43 and p-Cx43.According to the purpose of our experiment, we analyze the statistical difference between several certain groups. The levels of Cx43 and p-Cx43 of the NT group were calculated by the relative gray value; the Cx43 and p-Cx43 level of remaining groups were divided by the corresponding levels of Cx43 and p-Cx43 in NT group, then calculate the “relative expression” of these two proteins. The Cx43 and p-Cx43 “relative expression” levels of NT group are 1. Ischemic preconditioning (IP) significantly attenuated the expression of Cx43 at all re-oxygenation time points, including 1, 3, and 6 h. Compared with the normal group, oxygen-glucose deprivation/re-oxygenation (OGD/R) significantly increased the expression of p-Cx43, whereas IP counteracted this tendency at 1 and 3 h after re-oxygenation; at 6 h after re-oxygenation, IP increased the expression of p-Cx43. **c** Representative deconvolution fluorescent images of neuron-astrocyte co-cultures. All scale bars represent 100 μm. Neurons were stained using an anti-microtubule-associated protein II (MAP II) antibody (green). Astrocytes were stained using an anti-glial fibrillary acidic protein (GFAP) antibody (red). **d** Neuronal survival rate was quantified. The viability of non-treated neurons (NTs) was normalized to 1.0. Each column shows the mean ± standard error of the mean (*n* = 5). ***P < 0.001 when compared with the survival rate of non-treated co-cultures

As GJs are composed of Cx43, we first investigated the expression of Cx43 in several astrocyte culture conditions [no treatment (NT), OGD/R, IP + OGD/R at different time points (1, 3, and 6 h). In this part of the experiment, we obtained interesting experimental results relative to IP. In cultures subjected to OGD/R alone, Cx43 expression was variable at different time points, compared to NT cultures; in contrast, the expression of phosphorylated Cx43 (p-Cx43) was upregulated. IP also had an impact on the expression of Cx43 and p-Cx43. Cultures subjected to IP prior to OGD/R exhibited significantly decreased Cx43 expression and increased p-Cx43 expression (Fig. 1a, b). Thus, IP may participate in the regulation of Cx43 during the process of cerebral ischemia.

We then compared the number of surviving neurons (identified via MAP-II staining) and astrocytes (identified via GFAP staining) in various neuron-astrocyte culture conditions (NT, OGD/R, IP + OGD/R, IP + OGD/R + CBX) and at different time points following re-oxygenation (1, 3, and 6 h; Fig. 1c, d). Following 1 or 3 h of re-oxygenation, we observed no significant differences in the number of neurons among the IP + OGD/R, IP + OGD/R + CBX, and control conditions. This finding indicates that CBX and IP treatments exerted no beneficial effects upon cell survival in neuron-astrocyte co-cultures at these time points. However, after 6 h of re-oxygenation, significant differences were observed among groups: IP exerted protective effects against morphological damage and against cell death. In the OGD/R group, neurons disintegrated, protuberances diminished or disappeared, and the network structure was destroyed, whereas in the IP + OGD/R and IP + OGD/R + CBX groups, the morphological damage was attenuated. Furthermore, the neuronal survival rate at the 6-h time point was significantly higher in the IP + OGD/R + CBX than in the IP + OGD/R group. This finding indicates that treatment with CBX significantly enhanced the protective effects of IP during the re-oxygenation period following OGD.

**Fig. 2:**
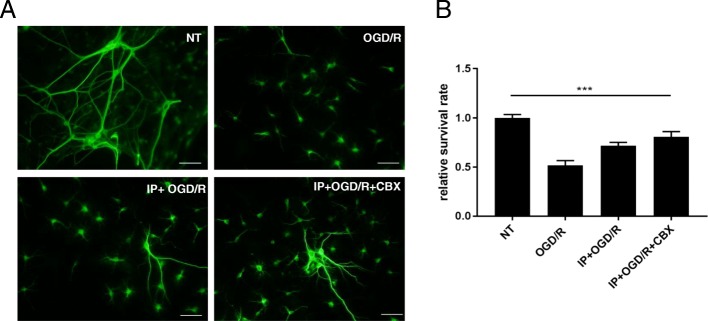
The effect of CBX on neurons. **a** Representative deconvolution fluorescence images of neuron cultures. Neurons were stained with an anti-MAP-2 antibody (green). Neuron cultures were treated with OGD/R, OGD/R with preconditioning, and OGD/R with CBX and IP, at 6 h after re-oxygenation. Scale bar = 50 μm. **b** Neuronal survival rate was quantified. The viability of non-treated neurons (NT) was normalized to 1.0. Each column shows the mean ± SEM (*n* = 5). ***P < 0.001 compared to the survival rate of non-treated neuron cultures

We next examined the direct toxic effect of OGD/R, OGD/R with preconditioning, and OGD/R with IP and CBX at 6 h after re-oxygenation on neuronal cells. Neuronal cell death was not induced by these treatments (Fig. 2a, b).

### Protective effects of CBX are associated with reductions in glutamate and ROS levels rather than of inflammatory factors

**Fig. 3:**
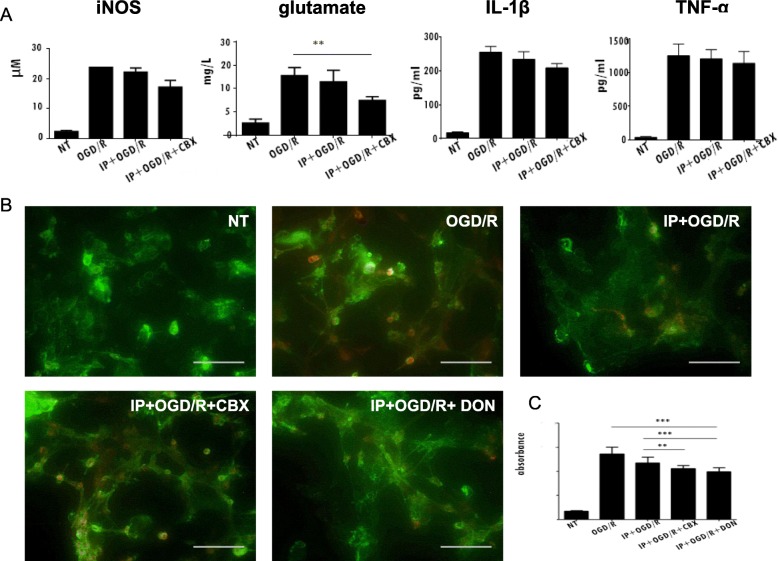
Levels of glutamate, reactive oxygen species, and inflammatory factors are affected by oxygen-glucose deprivation. **a** Cytokine levels. We used an enzyme-linked immunosorbent assay to measure interleukin 1 beta (IL-1β), tumor necrosis factor alpha (TNF-α), and inducible nitric oxide synthase (iNOS) levels, while levels of glutamate were measured using a colorimetric method. These measurements were obtained 6 h following oxygen-glucose deprivation (OGD/R). **b** Measurement of reactive oxygen species (ROS). Live cell staining for ROS was performed in astrocytes using the ROS sensor reagent CellROX™ Deep Red (red). The astrocyte cell surface marker anti-glutamate-aspartate transporter (GLAST, green) was also used. The images were obtained using deconvolution fluorescence microscopy. All scale bars represent 200 μm. **c** Measurement of absorbance. We also quantitatively measured ROS expression by collecting absorbance data using a microplate reader at a wave length of 485 nm. IP + 6-diazo-5-oxo-L-norleucine (DON) significantly reduced ROS relative to OGD/R and IP + OGD/R + CBX; IP + OGD/R, compared with OGD/R, exhibited weak statistical for a reduction in ROS. Compared with OGD/R, CBX, or DON reduced the ROS amount of OGD/R significantly. (**P < 0.01; ***P < 0.0001; each scale bar represents 200 μm)

We next investigated CBX effects on glutamate levels, a ROS-related indicator. Six hours following the reintroduction of oxygen and glucose, we measured the levels of glutamate, IL-1β, TNF-α, and NO. NO is generated by activated microglia and astrocytes [30] after stroke and is considered to be related to brain injury and neurotoxicity [31]. Furthermore, activated astrocytes can produce an array of pro-inflammatory cytokines, including IL-1β and TNF-α, which promote inflammation after stroke [32]. We found that IP significantly reduced the production of glutamate following OGD/R; however, no significant reductions in the levels of inflammatory factors were observed in the IP + OGD/R-treated cultures. Moreover, the levels of glutamate excitotoxicity were significantly lower in IP + OGD/R + CBX than in IP + OGD/R cultures, suggesting that CBX enhances the ability of IP to decrease excitotoxicity by reducing glutamate levels (Fig. 3a).

After cerebral ischemia reperfusion, glutamate is released in large quantities, thus contributing to the sharp rise of the concentration of extracellular glutamate and the activation of *N*-methyl-d-aspartate receptors, as well as the increase in mitochondrial ROS generation [33]. Moreover, Hu and Chen suggested that IP could decrease the levels of glutamate release and ROS generation [34]. Thus, we measured ROS levels via live cell staining using deconvolution fluorescence microscopy (Fig. 3b) and performed quantitative analysis of ROS expression by collecting absorbance data using a microplate reader (Fig. 3c). We found that ROS levels were significantly lower in the IP + OGD/R + CBX condition than in other conditions. Taken together, our results indicate that CBX might reduce the production of ROS by decreasing glutamate production in astrocytes; this might lower excitotoxicity in neurons following OGD/R, thereby enhancing the protective effects of IP.

### Inhibition of glutamate synthesis and ROS production but not of inflammatory factors leads to effective neuroprotection

**Fig. 4:**
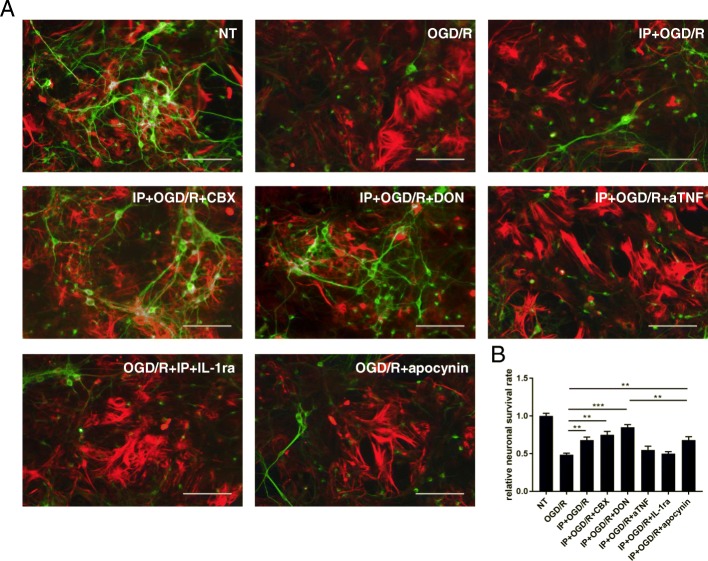
The effects on neuronal survival following inhibition of the synthesis of glutamate, reactive oxygen species, and inflammatory factors. Following 6 h of re-oxygenation, we added 6-diazo-5-oxo-l-norleucine (DON), anti-TNF-α antibody (aTNF), anti-IL-1β receptor antibody (IL-1ra), and apocynin to neuron-astrocyte co-cultures that were treated with oxygen-glucose deprivation (OGD) to ensure blockade of the synthesis of glutamate, TNF-α, IL-1β, and reactive oxygen species (ROS), respectively. We assessed alterations in cell numbers in the four groups at 1, 3, and 6 h after OGD. **a** Representative deconvolution fluorescence images of neuron-astrocyte co-cultures. Neurons were stained with an anti-microtubule associated protein II (MAP-II) antibody (green). Astrocytes were stained with an anti-glial fibrillary acidic protein (GFAP) antibody (red). All scale bars represent 100 μm. **b** The relative neuronal survival rate was quantified. The viability of non-treated neurons was normalized to 1.0. Survival rate of the remaining groups divided by the survival rate of NT group to calculate the relative survival rate. To assess the effect of IP and various drugs on neuronal survival after OGD/R, we calculated the statistical differences between each drug intervention group and the OGD/R group, respectively. Each column indicates the mean ± SEM (*n* = 5). ***P < 0.001 when compared to the survival rate of OGD/R co-cultures

Following 6 h of re-oxygenation, we added DON, commonly used for inhibiting Gls to reduce glutamate production [29], aTNF, IL-1ra, or apocynin [NADPH (nicotinamide adenine dinucleotide phosphate) oxidase inhibitor] to neuron-astrocyte co-cultures that were treated with OGD to ensure the blockade of glutamate, TNF-α, IL-1β, and ROS synthesis, respectively, in neurons. Apocynin is a type of NADPH oxidase inhibitor; notably, Lu et al. confirmed an important role for NADPH oxidase-derived superoxide in oxidative stress associated with OGD [35]. Treatment with DON or CBX significantly inhibited excitotoxicity and enhanced the survival of neurons, while treatment with apocynin resulted in partial rescue of neuronal survival. However, treatment with 10 μg/mL of either aTNF or IL-1ra produced no significant decrease in cell death following OGD/R (Fig. 4a, b).

### IP combined with CBX effectively blocks Cx43 GJs and attenuates Gls gene expression and EAAT levels in astrocytes

**Fig. 5:**
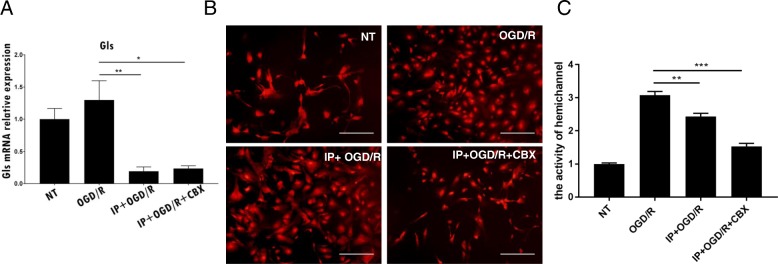
Ischemic preconditioning attenuated levels of functional Cx43 gap junction hemichannels and glutaminase mRNA expression in astrocytes. **a** Real-time quantitative PCR results. The relative expression of glutaminase (Gls) mRNA in all samples was quantified in comparison with glyceraldehyde 3-phosphate dehydrogenase (GAPDH). Oxygen-glucose deprivation/re-oxygenation (OGD/R) increased Gls mRNA expression in astrocytes, but ischemic preconditioning (IP) and IP + carbenoxolone (CBX) reduced the expression of Gls mRNA. **b** Fluorescence microscopy shows that both IP alone and IP + CBX attenuate the function of Cx43 hemichannels at the cell surface of astrocytes. All scale bars represent 200 μm. **c** Quantification of the function of Cx43 hemichannels. NT, non-treated

It was previously demonstrated that IP or CBX decrease glutamate levels in cell cultures after OGD/R and that Gls is an important enzyme involved in glutamate production [36]. We therefore investigated whether CBX influences *Gls* mRNA expression levels or blocks the release of glutamate. Real-time PCR analyses revealed that the expression of *Gls* mRNA was lower in cultures subjected to IP + CBX than to OGD/R (Fig. 5a). Based on this finding, we examined the function of hemichannels in each condition by assessing Etd uptake. We observed that Cx43 hemichannels were functionally occluded by CBX exposure. The above results indicate that glutamate is released by Cx43 hemichannels at GJs and that blockade of GJs reduces glutamate synthesis in astrocytes (Fig. 5b, c).

**Fig. 6:**
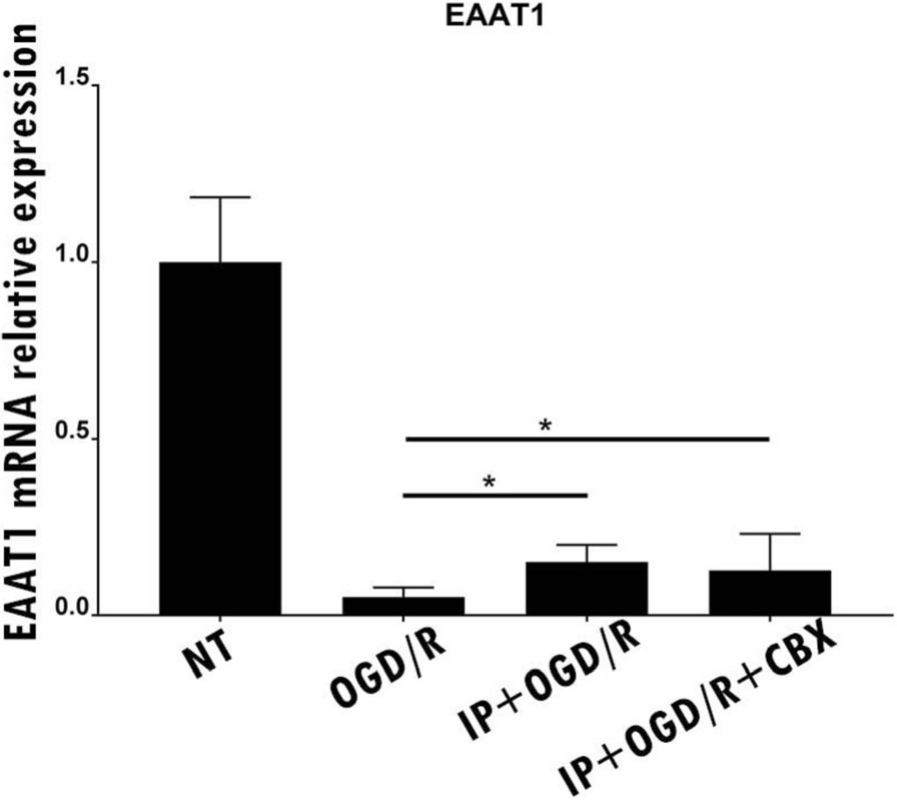
Ischemic preconditioning (IP) and IP + carbenoxolone increased efficient excitatory amino acid transporter 1 expression in astrocytes following oxygen-glucose deprivation/re-oxygenation. Real time PCR for excitatory amino acid transporter 1 (EAAT1) mRNA. The relative expression of EAAT1 mRNA in all samples was quantified in comparison with glyceraldehyde 3-phosphate dehydrogenase (GAPDH). Levels of EAAT1 mRNA expression were reduced following oxygen-glucose deprivation/re-oxygenation (OGD/R). However, this trend is mitigated by ischemic preconditioning (IP) and IP + carbenoxolone (CBX)

We then investigated whether CBX influenced EAAT expression levels by performing Western blot analysis. We found that EAAT1 was upregulated in cultures subjected to IP and IP + CBX (Fig. 6). Taken together, these findings suggest that the occlusion of hemichannels by CBX affects both the release and clearance of glutamate.

## Discussion

IP refers to the administration of a non-lethal transient ischemic stimulus prior to the onset of a lethal ischemic stroke; this triggers a collective endogenous protective mechanism [1–6]. Cx43 is a connexin extensively found on the astrocytic surface and consists of six protein subunits, which are embedded in the plasma membrane and form a hemichannel that enables the exchange of small molecular substances and information transfer in and out of the cell [37]. We thus explored the mechanism by which preconditioning attenuates the neurological impairment caused by ischemic stroke and investigated whether preconditioning exerts neuroprotective effects by modulating the activity of Cx43 hemichannels or whether additional protective mechanisms are in play. Our experiments demonstrated that OGD/R results in glutamate release and ROS production, which contribute to cell injury. The use of CBX to block GJs reduces the levels of glutamate and ROS, thus protecting cells, while IP enhances these protective effects.

We used CBX and treated cultures for specific time-points, selected based on our previous study [27], i.e., at 1, 3, and 6 h following re-oxygenation. Our results showed that combination of IP and CBX produce the most dramatic effects at the 6-h time point. In terms of neuronal survival rate, a statistical difference between IP and drug protection was observed at 6 h, but not at 1 or 3 h. The mechanism behind this difference is not clear; however, it would be interesting to examine whether IP and CBX would exert a protective role for longer periods of reoxygenation. Further research in our laboratory is underway towards this direction.

In the present study, we observed significant increases in neuronal survival and morphological preservation following 6 h of re-oxygenation in neuron-astrocyte co-cultures that were subjected to IP prior to OGD, relative to controls. Furthermore, these effects were significantly enhanced by the addition of CBX to cultures that were subjected to IP, prior to OGD. These results indicate that CBX enhances the protective effects of IP during the re-oxygenation period following OGD. Additional experiments revealed that 6 h following re-oxygenation, astrocytes-neurons co-cultures subjected to IP exhibited significantly decreased the production of glutamate. These effects were significantly enhanced by the CBX treatment, suggesting that CBX increases the ability of IP to decrease excitotoxicity by reducing glutamate levels. However, no significant reductions in the levels of inflammatory factors or ROS production were observed. This may be because the main players in the inflammatory response in the central nervous system are microglia; after ischemic stroke, microglia are rapidly activated, performing phagocytosis of necrotic cells and producing a variety of inflammatory factors [38]. In addition to microglia, astrocytes also exhibit potent pro-inflammatory potential when stimulated [19]. However, in our experiments, we found that blockade of astrocyte hemichannels by CBX did not affect the level of cytokines produced. However, we used aTNF and IL-1ra at a final concentration of 10 μg/mL [39]. Therefore, further studies are required to determine whether this difference in concentration might explain our experimental results.

In the present study, we treated astrocyte-neuron co-cultures with DON, the most commonly used Gls inhibitor [29], to determine whether lethal doses of ROS were associated with glutamate levels. Our results showed that CBX partially reduces ROS generation, inhibits additional glutamate release from astrocytes, rescues neurons in the co-culture system following OGD, and enhances the protective effects of IP. And we also confirmed that CBX could inhibit the release of glutamate compared with DON, which means CBX could reduce part of the ROS. Moreover, the inhibition of excitotoxicity by DON or CBX—rather than the inhibition of inflammatory factors—significantly increases the survival rate of neurons, in accordance with the findings of previous studies [19, 21].

**Fig. 7:**
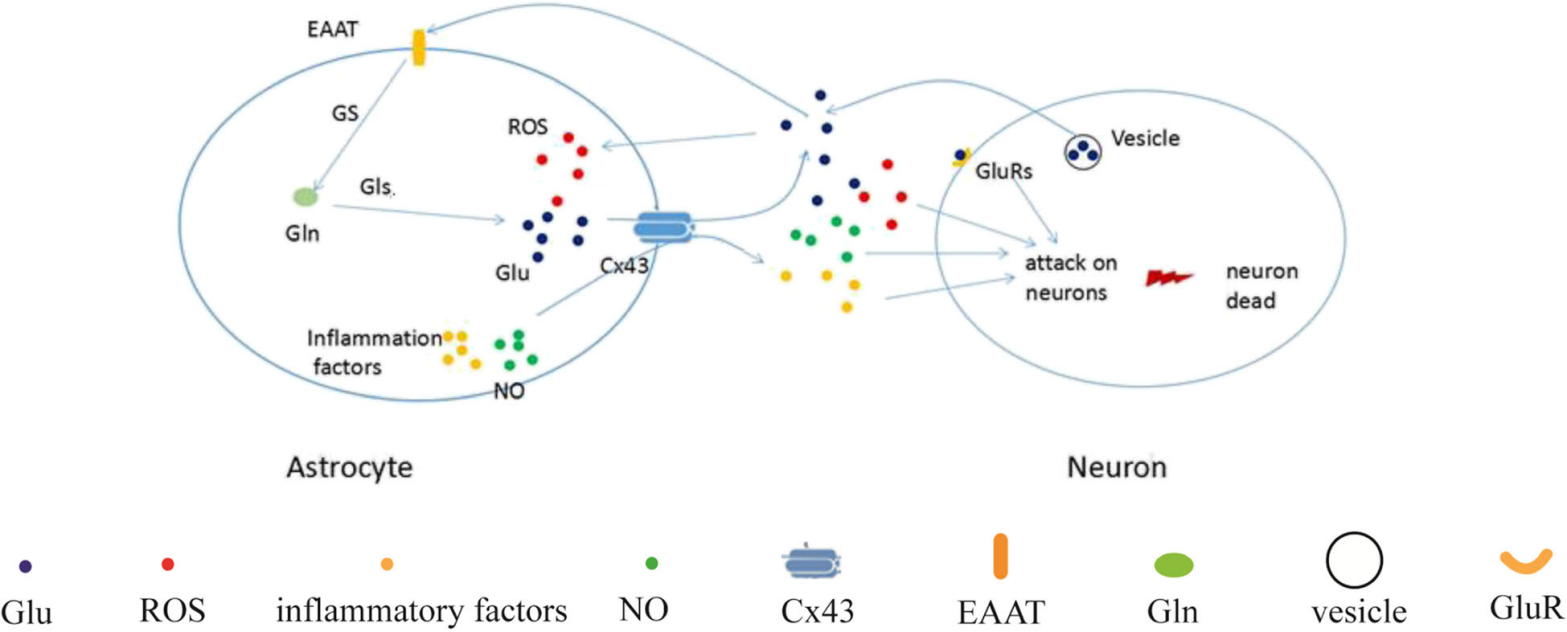
Function and regulation of connexin 43 in astrocyte-neuron co-cultures. The attack of ischemia results in massive vesicular release of glutamate from neurons. The astrocyte glutamate transporter, EAAT, transport extracelluar glutamate into the cell, downregulate extracellular glutamate level. The glutamate entering astrocytes were synthesized to glutamine under the catalysis of glutamine synthetase, which can be hydrolysis to glutamate by the action of glutaminase. After the attack of ischemia, the expression and function of EAAT is decreased, resulted in reduced glutamate clearance; excessive glutamate promotes the production of ROS; meanwhile, astrocyte stimulation produce inflammatory factors, all of them cause neuronal death. Cx43, connexin 43; EAAT, excitatory amino acid transporter; NO, nitric oxide synthase; ROS, reactive oxygen species; Gls,glutaminase; GluRs, glutamate receptors; GS, glutamine synthetase; Gln, glutamine; Glu, glutamate.

Our results further indicated that, while glutamine levels are upregulated by OGD/R, they remain unaffected by either IP or CBX, suggesting that the protective effects of IP/CBX are not due to alterations in glutamate biosynthesis. We then examined the function of GJs in each culture condition. Etd uptake was significantly higher in the OGD group than in the no treatment group, and this effect was significantly blocked by CBX. Taken together, these findings confirm the successful inhibition of GJs by CBX treatment and demonstrate that CBX effectively reduces the release of glutamate without affecting its biosynthesis (Fig. 7).

Glutamate is released by different cells, including neurons, astrocytes, and microglia. After cerebral ischemia, as a result of calcium overload, presynaptic neurons release a large amount of glutamate, stored in the vesicles. Glial cells also participate in this release, upon stimulation by microglia. Several studies have reported the release of glutamate by astrocytes after ischemic stroke [40]. In our study, CBX blockade of astrocyte hemichannels after OGD/R effectively reduced glutamate levels in the astrocytes and neurons co-culture systems, suggesting that OGD/R promote the release of glutamate through the hemichannels of astrocytes.

EAATs are membrane-bound secondary glutamate transporters, which superficially resemble ion channels [41]. In the brain, EAATs remove glutamate from the synaptic cleft and extrasynaptic sites by promoting glutamate reuptake into glial cells and neurons, thus regulating the concentration of glutamate in the extracellular space [42]. Following an action potential, glutamate transporters quickly remove glutamate from the extracellular space, thereby terminating synaptic transmission [41, 43]. Subtypes of EAAT1 are found in the membranes of astrocytes; EAAT1 is also abundant in the cerebellum and forebrain [14]. In the present study, we observed that expression of EAAT1 was reduced following OGD/R, upregulated following IP, and further increased when cultures subjected to IP were treated with CBX. Nevertheless, further studies are required to more fully elucidate the expression and distribution of EAAT1 following cerebral ischemia.

Our results appear to be consistent with most previous studies. For example, Takaki et al. found that increased concentrations of extracellular glutamate downregulate glutamate transporters on astrocytes [44]. Our findings may provide a reasonable explanation. CBX and IP reduce glutamate concentration in the cell culture system after OGD/R, thereby reducing the inhibition of glutamate transporter expression on astrocytes. However, there are also some reports which are inconsistent with our findings. In an in vivo experiment, Nakase et al. [23] found that, 4 days post-stroke, there is a larger stroke volume, as well as increased apoptosis and inflammation, in mice lacking Cx43 in astrocytes (Cx43^(fl/fl)^:hGFAPcre mice), which is largely contradictory to our results. However, the authors in that study used a permanent middle cerebral artery occlusion model, which mimics the lack of cerebral blood flow re-establishment after stroke, whereas, in our study, we explored the function of Cx43 in the phase of reperfusion. Since the mechanisms governing the phase of ischemia and reperfusion are not the same, animal models of stroke have been divided into permanent and transient ones. During the ischemic phase, nerve damage is caused due to lack of oxygen and glucose, calcium overload, and cell necrosis, termed as primary injury; in the reperfusion phase, cell death is caused by secondary attack through various molecular mechanisms, including the generation of ROS and various molecular cascade reactions, via mechanisms that are different from those of primary injury [24]. Thus, it is possible that Cx43 plays different roles in these two different stages. Further, the expression level of Cx30 in astrocytes of Cx43^(fl/fl)^:hGFAPcre mice was elevated [25]; thus, the increased post-stroke severity observed in these mice may not be directly associated with the absence of Cx43. Moreover, whether Cx43 gene knockout mice exhibit other physiological changes is not known.

In the absence of glutamate transporter activity, glutamate builds up and leads to cell death due to excitotoxicity, since excessive glutamate acts as a toxin to neurons by triggering a number of biochemical cascades. The activity of glutamate transporters also allows glutamate to be recycled for repeated release [6]. In order to explore the mechanisms by which CBX attenuates such damage, we exposed neuron-astrocyte co-cultures to IP or IP + CBX, following OGD/R. Previous studies have demonstrated that IP significantly reduces ischemia-reperfusion damage by reducing the levels of inflammatory factors [1–6]. Although we observed no significant effects of CBX on the levels of inflammatory factors, previous research suggests that neuroinflammation is closely associated with the release of ROS and glutamate [45]. Therefore, future studies should aim to determine whether GJs are involved in secondary damage following inflammation due to cerebral ischemia reperfusion.

GJs play an important role in the transmission of neural impulses due to electrical and metabolic coupling, which allow small metabolites and signaling molecules (e.g., cyclic adenosine monophosphate and Ca^2+^) to shuttle between the connected cells without contacting the extracellular space. Therefore, when some of these connected cells receive such signals, the entire cell population can respond. Although our findings indicate that CBX inhibits GJs during IP, further studies are required to determine whether alterations in Cx43 influence the levels of inflammation following ischemia.

In the myocardial ischemia model, Cx43 deficiency increases myocardial tolerance to ischemia-reperfusion injury and eliminates the protective effect of preconditioning [46], which means that in this model, Cx43 may be involved in the protective mechanism of preconditioning. However, whether Cx43 plays a role in the protective mechanism of preconditioning remains to be explored in cerebral ischemia model.

GJs connect the cytoplasm of adjacent cells, allowing communication between cells and exchange of small molecules. GJ connections between astrocytes are commonly found in the brain and play a role in brain development, including cell differentiation, migration, and survival. In contrast, they exert pathophysiological effects in human diseases, such as ischemic stroke. Thus, GJs can be viewed as a “double-edged sword.” Farahani et al. [47] suggested that GJs may be beneficial after injury. Cell damage leads to the accumulation of harmful metabolites in the cytoplasm, such as calcium, glutamate, potassium, and reactive oxygen species [48], and is a key factor in excitotoxicity. GJ communication allows these factors to move through adjacent healthy cells. By allowing healthy cells to buffer such toxic metabolites, damaged cells can be rescued from dying and can secrete various neurotrophic factors and cytokines that stimulate the survival of neighboring neurons, thereby protecting them from excitotoxic, metabolic, and oxidative damage. Conversely, harmful substances can also flow from one cell to another, damaging otherwise healthy and functioning cells, causing more cellular damage, (e.g., by allowing death messengers to spread from damaged cells to other healthy adjacent cells, thus extending the lesion area). Therefore, the study of the role of GJs in stroke may provide a clinical breakthrough of great significance.

## Conclusions

In summary, our findings demonstrate that IP blocks GJs between astrocytes and reduces the extracellular glutamate content. In hypoxic environments, GJs of astrocytes release glutamate, leading to excitotoxicity. Moreover, our results indicate that administration of CBX decreases ROS production and excitotoxicity by reducing glutamate levels following OGD/R and that such effects enhance the overall neuroprotective functions of IP (Fig. 7). However, blocking of inflammatory factors produced by astrocytes, such as IL-1β and TNF-α, does not produce any significant effect on neuronal survival. Taken together, our findings suggest that CBX intervention can enhance the protective effects of IP at GJs and reduce neuronal damage, by decreasing the release of glutamate and ROS in astrocytes.

## Abbreviations

aTNF: Anti-TNF-α antibody
CBX: Carbenoxolone
Cx43: Connexin 43
DON: 6-Diazo-5-oxo-l-norleucine
E17: Embryonic day 17
EAAT: Excitatory amino acid transporters
GADPH: Glyceraldehyde 3-phosphate dehydrogenase
GFAP: Glial fibrillary acidic protein
GJ: Gap junction
GLAST: Glutamate-aspartate transporter
IL-1ra: Anti-IL-1β receptor antibody
IL-1β: Interleukin-1β
IP: Ischemic preconditioning
MAP-II: Microtubule-associated protein II
mtCx43: Mitochondrial Cx43
NADPH: Nicotinamide adenine dinucleotide phosphate
NO: Nitric oxide
NT: No treatment
OGD: Oxygen-glucose deprivation
p-Cx43: Phosphorylated Cx43
PEI: Polyethyleneimine
ROS: Reactive oxygen species
TNF-α: Tumor necrosis factor α
